# TRPM2 in the Brain: Role in Health and Disease

**DOI:** 10.3390/cells7070082

**Published:** 2018-07-22

**Authors:** Giulia Sita, Patrizia Hrelia, Agnese Graziosi, Gloria Ravegnini, Fabiana Morroni

**Affiliations:** Department of Pharmacy and Biotechnology, Alma Mater Studiorum, University of Bologna, Via Irnerio 48, 40126 Bologna, Italy; giulia.sita2@unibo.it (G.S.); agnese.graziosi2@unibo.it (A.G.); gloria.ravegnini2@unibo.it (G.R.); fabiana.morroni@unibo.it (F.M.)

**Keywords:** TRPM2, brain, aging, neurodegeneration, neurodegenerative disease

## Abstract

Transient receptor potential (TRP) proteins have been implicated in several cell functions as non-selective cation channels, with about 30 different mammalian TRP channels having been recognized. Among them, TRP-melastatin 2 (TRPM2) is particularly involved in the response to oxidative stress and inflammation, while its activity depends on the presence of intracellular calcium (Ca^2+^). TRPM2 is involved in several physiological and pathological processes in the brain through the modulation of multiple signaling pathways. The aim of the present review is to provide a brief summary of the current insights of TRPM2 role in health and disease to focalize our attention on future potential neuroprotective strategies.

## 1. Introduction

Transient receptor potential (TRP) proteins form non-selective cation channels which are involved in several cell functions in activated form. To date, about 30 different mammalian TRP channels have been recognized, which are divided into six subfamilies: TRPA (Ankyrin), TRPC (canonical), TRPM (melastatin), TRPML (mucolipin), TRPP (polycystin) and TRPV (vanilloid) [1]. Most TRP channels have a role in sensory perception in animals and they all share structural similarities [2]. Indeed, they contain six transmembrane regions with the C- and N-termini located intracellularly. Furthermore, they function mostly as heterotetramers or homotetramers that form a central ion permeation path between the fifth and sixth regions [3]. These channels are non-selective polymodal cation channels that are located in the plasma membrane. Their stimulation produces cell depolarization, which leads to the opening or closing of voltage-dependent ion channels and subsequently, affects the modulation of the ion cellular flux. TRPs are mainly calcium (Ca^2+^) release channels that are found in several cell organelles, such as lysosomes, endosomes, endoplasmic reticulum, synaptic vesicles and the Golgi network [4]. As polymodal channels, TRPs can be activated by different physical and chemical stimuli, such as mechanical forces, temperature, intracellular signaling molecules and exogenous compounds. However, to date, few endogenous ligands are recognized as “TRP activators” [5,6,7]. There are three different ways of activation for these channels: activation by receptor, ligand activation and direct activation [7]. In the first case, G protein–coupled receptors (GPCRs) and receptor tyrosine kinases activate phospholipases C (PLCs) that can regulate channel activity by the hydrolysis of phosphatidylinositol bisphosphate (PIP2) or by the production of diacylglycerol (DAG) or inositol trisphosphate (IP3), which results in the liberation of intracellular Ca^2+^ [8,9,10]. Ligands that activate TRP channels may be exogenous or endogenous small organic molecules; purine nucleotides and their metabolites (adenosine diphosphoribose (ADP-ribose), βNAD+); or inorganic ions, especially Ca^2+^ and magnesium (Mg^2+^). Since Ca^2+^ is a key excitatory messenger between neurons [11], its intracellular levels are finely regulated and an excessive load can lead to metabolic instabilities and in the worst case, cell death [12,13]. Finally, direct activation includes channel phosphorylation, mechanical stimuli and conformational coupling to IP3 receptors. Moreover, TRP channels can be indirectly activated by heating and cell swelling through second messengers or other unknown mechanisms [14].

The TRPM family is composed of TRPM1/M3, TRPM4/M5, TRPM6/M7, TRPM2 and TRPM8 [15]. These channels show a TRP segment at the C-terminal transmembrane domain that can be divided in two regions: a coiled-coils domain, which participates in the assembly of the channel into its tetrameric form and a second variable region. The coiled-coils domain is a structural motif in proteins, which allows the formation of α-helices that zip up together in a helical coil conformation [16]. Coiled-coils are identified in protein sequences by their characteristic repetition of aliphatic residues that alternates every few residues to form seven residue reappearances. Although the sequence patterns are an expression of three-dimensional coiled-coil structures, the resulting assembly cannot be accurately predicted [2]. TRPM proteins are implicated in a plethora of physiological mechanisms. In particular, TRPM2 is involved in the response to oxidative stress.

In the brain, TRPM2 is the most abundant TRP channel [17]. As a newly identified non-selective Ca^2+^-permeable cation channel and a sensor of reactive oxygen species (ROS), TRPM2 channel has recently been indicated to be involved in several physiological and pathological processes of the central nervous system (CNS) through the modulation of multiple signaling pathways.

In this review, after a brief insight in the main features of TRPM2, we focused on the role of this protein in aging and in common chronic and acute neurodegenerative diseases.

## 2. TRPM2 inside the Brain

**Figure 1:**
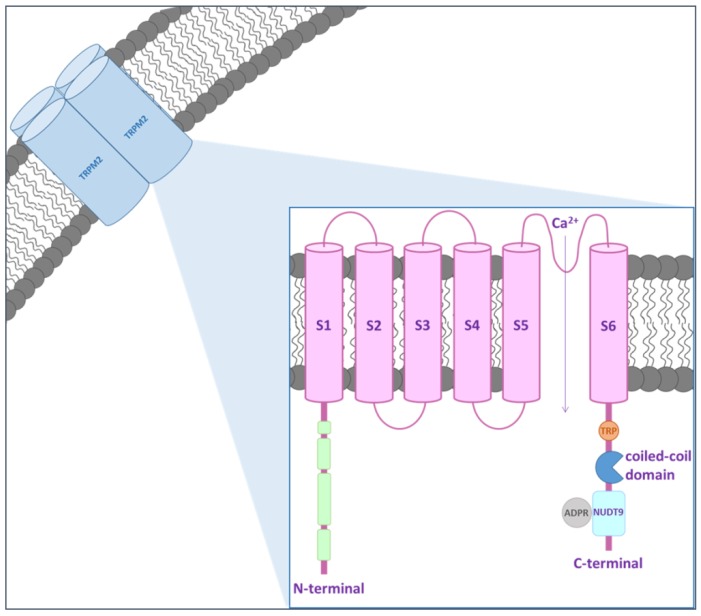
TRPM2 protein structure. TRPM2 channel includes 6 transmembrane domains, with a re-entry loop that forms a pore located between domains 5 and 6. Regulatory regions of the channel are contained within the cytoplasmic N- and C-termini.

TRPM2 channel was described for the first time in 1998 by Nagamine et al. [18]. This protein is a Ca^2+^-permeable non-selective cation channel without voltage-dependent behavior. The molecular basis for the permeability of TRPM2 to divalent cations, such as Ca^2+^ and Mg^2+^, is still not fully understood but it seems to be regulated by amino acid residues located between the pore helix and the selectivity filter in the channel pore [19,20]. It is widely expressed, especially in the CNS, heart, lung, liver and pancreas [17,21,22]. Recently, Zhang et al. [23] described the structure of TRPM2 channel from *Nematostella vectersis*, especially when bound to Ca^2+^, by electron cryo-microscopy (cryo-EM). They demonstrated that the structure of TRPM2 differs in local geometry and surface polarity from the other TRPM family channels, which may explain many of its unique functional features. In particular, the larger pore diameter and the negative surface charge of pores may contribute to determining its higher Ca^2+^ permeability compared to TRPM4 [23]. Within the brain, TRPM2 is expressed in microglia, astrocytes and neurons of the hippocampus, substantia nigra, striatum and cortex [15,24]. Due to its ubiquitous distribution, the channel may be involved in several physiological processes. The intracellular Ca^2+^ is a driving force for TRPM2 activity, which also depends on the presence of extracellular hydrogen peroxide (H_2_O_2_) [25,26]. TRPM2 is composed by a nudix hydrolase domain that is highly homologous to the ADP pyrophosphatase, NUDT9 [27,28] (Figure 1). Several extracellular stimuli, including ROS, H_2_O_2_, amyloid β-peptide (Aβ) and tumor necrosis factor-α (TNF-α), have been shown to induce TRPM2 activation via metabolic production of intracellular poly ADP-ribose (ADPR) polymerase, which is an enzyme that transfers multiple ADPR groups to proteins [25,29,30]. The inhibitors of polyADPR polymerase prevent TRPM2 activation without blocking the channel directly [30].

TRPM2 is expressed in both neurons and glia and it contributes to hippocampal synaptic plasticity [31,32]. In 2011, Xie et al. [32] showed that the long term depression (LTD) is selectively impaired in TRPM2^−/−^ mice because of the inhibition of the glycogen synthase kinase 3β (GSK3β) and decreased postsynaptic density protein 95 (PSD95). TRPM2 is also involved in neuronal development and plays an inhibitory role in neurite outgrowth. Indeed, the inhibition of TRPM2 increases the axonal growth [15,33]. In light of this, TRPM2^−/−^ mice show longer neurites and more spines than wild type animals. Moreover, TRPM2 appears to be responsible for the physiological activation of the microglia mediated by ROS and (lipopolysaccharide) LPS signaling, while it is also involved in nitric oxide (NO) production [34,35]. Indeed, as suggested by studies involving TRPM2^−/−^ mice, the channel’s sensitivity to H_2_O_2_ depends on the activation state of the microglia [36]. In 2014, Miyake et al. [37] demonstrated in cultured microglia that combined treatment with LPS and interferon-γ (IFNγ) can result in TRPM2-mediated extracellular Ca^2+^ influx. This signal is absent after pharmacological blockade or gene deletion of TRPM2 channel, while p38 mitogen-activated protein kinases (p38MAPK) and c-Jun N-terminal kinases (JNK) signaling contribute to the activation of microglia induced by LPS or IFNγ [37].

## 3. TRPM2 outside the Brain

In addition to CNS expression, TRPM2 is also localized in different cell types of the peripheral immune system, including neutrophils [38,39], macrophages [40,41], lymphocytes [42,43], monocytes [44] and dendritic cells [45]. Following an injury, Ca^2+^ influx through TRPM2 can directly mediate cytokine release, which contributes to the recruitment and activation of the inflammatory response [46]. Interestingly, in 2011, Knowles et al. [47] demonstrated that TRPM2-deficient mice showed low levels of cytokines, IL-12 and IFNγ and consequently, were more susceptible to infection. As shown by Di et al. [48], TRPM2 can also inhibit ROS production and prevent endotoxin-induced inflammation in phagocytic cells. Indeed, the depolarization of the plasma membrane causes a decrease in ROS levels mediated by (nicotinamide adenine dinucleotide phosphate) NADPH oxidase. As a result, TRPM2^−/−^ mice that were exposed to endotoxins demonstrated augmented inflammatory responses and decreased survival compared to wild type animals. Moreover, Kashio et al. proved that TRPM2 may be involved in the phagocytic activity enhanced by fever in mice peritoneal macrophages [49]. To summarize, TRPM2 can be considered as a H_2_O_2_-activated cation channel that is involved in the host-defense system of the body.

Outside of the immune context, functional TRPM2 is also expressed in pancreatic β-cells where its activity contributes to the regulation of insulin secretion [49,50]. Insulin is secreted by pancreatic islets to control blood glucose levels and the downregulation of TRPM2 diminishes glucose-stimulated insulin secretion [51,52]. Recently, Uchida et al. [51] have shown both impaired glucose tolerance and decreased insulin secretion in TRPM2^−/−^ mice. Although plasma insulin levels were similar in wild type and TRPM2^−/−^ mice, the basal blood glucose levels were higher in transgenic animals than the others.

Moreover, the peculiarity of TRPM2 is its temperature sensitivity [49,50]. In recent years, Tan et al. demonstrated that TRPM2 is the ion channel responsible for the non-painful warmth sensitivity in somatosensory neurons, which they determined through a complex combination of electrophysiology, imaging, and RNA sequencing techniques [53,54]. In addition, as suggested in 2016 by Song et al., TRPM2 is also a hypothalamic temperature sensor. Indeed, the channel is able to limit the fever response and prevent overheating [55]. As Togashi et al. reported in 2006 [50], the activation of TRPM2 channel by heating was observed in HEK293T cells when the temperature ranges between 33 °C and 34 °C. However, the ion current that was evoked without any endogenous agonists was consistently lower than that in the presence of agonists, as confirmed by the same author in 2012 [49]. Although the temperature threshold for TRPM2 is around 47 °C, H_2_O_2_ treatment lowers the temperature needed for its activation to physiological temperature, showing that redox signals and temperature act as synergic stimuli [56,57]. In addition, TRPM2 is proposed to function in monocyte chemotaxis, which is known to be regulated by ADPR [58]. Nevertheless, TRPM2 channel is expressed throughout the body [17] and its role in Ca^2+^ homeostasis makes it an eligible candidate to mediate Ca^2+^-dependent physiological processes.

## 4. Role of TRPM2 in Brain Diseases

In developed countries, neurodegenerative diseases are considered as the most frequent cause of death after cancer and they have been predicted to surpass cancer as the most frequent cause before the year of 2040 [59]. Neurodegenerative diseases can be classified into two predominant types: chronic diseases (Alzheimer’s disease, AD; and Parkinson’s disease, PD) and acute diseases (cerebral ischemia, CI; and traumatic brain injury, TBI). They are all characterized by the presence of several common risk factors. In particular, oxidative stress, inflammation and aging play a crucial role in the evolution of these diseases [60]. It is important to understand that aging has numerous effects on the brain, including neurochemical changes, alterations in blood flow and reductions in white matter [61].

Oxidative stress results from an imbalance between the production of prooxidant and antioxidant agents, which are responsible for the rapid detoxification of the reactive intermediates on biological systems [62]. ROS production is not only physiological and occurs with respiration, but also increases in pathological transitory conditions (infections) or in more devastating diseases, such as chronic and acute neurodegenerative diseases, cancer, diabetes mellitus and autoimmune diseases [63,64]. Several studies suggested that the pathophysiology of neurodegenerative diseases is closely related to inflammatory responses and oxidative stress mediated by microglia [65,66,67]. Considering this, the role of oxidative stress may be considered as the most critical step in the treatment of these pathologies. It is worth mentioning that oxidative stress is also a feature of the physiological aging process. Furthermore, aging is responsible for neuronal Ca^2+^ dysregulation, reduction in antioxidant defense, increases in oxidative stress and perturbation of energy metabolism [68,69]. The involvement of oxidative stress during the aging process is widely accepted. Indeed, ROS increases with age and leads to functional alterations and pathological conditions [70]. Evidently, TRPM2 is thought to play a role in the aging process within the brain. Indeed, aging is related to decreased levels of glutathione (GSH) in vitro and in vivo [71]. GSH is the main intracellular reducing agent [72] and the first line of defense against ROS and reactive nitrogen species (RNS), which are generated as the by-products of aerobic metabolism. During aging, the cellular levels of GSH decrease and the resulting decline in antioxidant defense contributes to the increased susceptibility to age-related neurodegenerative diseases [73,74]. The depletion of GSH with age may also be associated with increased intracellular Ca^2+^ and its subsequent toxicity [75]. This process may involve TRPM2 as a recent study has demonstrated that the loss of inhibition by GSH enhances TRPM2 activity in hippocampal neurons [73]. As a consequence, the TRPM2 activity is associated with increased LTD [76] and these changes in synaptic plasticity produce an age-related decrease in synaptic strength, which may underlie the memory impairment associated with aging [20].

### 4.1. Chronic Neurodegenerative Diseases

Since TRPM2 is abundantly expressed in the CNS, it is not unexpected that this channel has been related to neurodegenerative diseases [77]. AD is the most frequent cause of senile dementia, which affects millions of people worldwide [78]. This disease is characterized by a progressive decline in cognitive function and in the final stage, patients are completely unable to carry out any daily activities. AD is characterized by a dramatic increase in oxidative stress and neuroinflammatory conditions, intracellular Ca^2+^ dysregulation and the presence of protein depositions, such as extracellular beta-amyloid (Aβ) plaques and intracellular tangles of tau protein [79]. Animals treated with Aβ show impaired learning and memory; activated astrocytes and microglia; and disturbed activation of c-JNK and GSK3β [80,81], which supports the role of glial cells in AD pathology. Activated astrocytes produce and release gamma-aminobutyric acid (GABA) by monoamine oxidase-B. By acting on presynaptic GABA receptors, the released GABA from astrocytes decreases the spike probability of granule cells in the dentate gyrus of AD model mice, which impairs the synaptic plasticity, such as learning and memory abilities [82]. One of the most accepted hypotheses implies that mitochondrial dysfunction and oxidative stress are the primary events in the onset of pathology [83]. Interestingly, different studies have observed that oxidative stress modifies Ca^2+^ homeostasis in AD patients and in animal models [84,85]. Indeed, accumulating evidence suggests that an overloaded intracellular free Ca^2+^ concentration increases mitochondrial membrane depolarization, oxidative stress and apoptotic cell death [86].

GSH has a physiological decrease in aging and a pathological decrease in AD [87]. To this end, primary neuronal cells may be cultured beyond 3 weeks to obtain characteristic cellular changes that have been associated with aging and neurodegeneration [88]. In these neurons, the increased current of TRPM2 channel can be decreased by GSH supplementation [73]. Furthermore, the depletion of GSH can induce oxidative stress, disturbance of Ca^2+^ homeostasis and apoptosis of hippocampal neurons through activation of TRPM2 channel [89].

Oxidative stress caused by the inhibition of GSH biosynthesis induces human microglia and astrocytes to secrete TNF-α, IL-6 and nitrite ions and to increase the concentration of intracellular Ca^2+^. These effects are correlated with the activation of inflammatory signaling of p38MAPK, JNK, and nuclear factor kappa-light-chain-enhancer of activated B cells (NFκB), which are reduced by the pharmacological blockade of TRPM2 channel or genetic ablation of its expression [21]. This confirms the contribution of TRPM2 channel to AD pathology [90].

**Figure 2:**
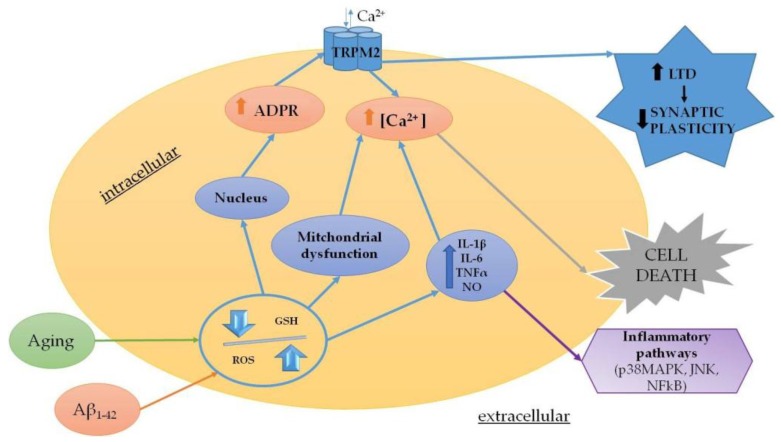
Summary of the main mechanisms modulated by TRPM2 in chronic neurodegenerative diseases.

Fonfria et al. exposed primary striatal cells to 20 μmol/L of monomeric Aβ to demonstrate an increase in intracellular Ca^2+^ and cell death, which were partially blocked when the cultures were transfected to inhibit TRPM2 functions [91]. More evidence of the contribution of TRPM2 to pathology and cognitive decline was recently provided in a transgenic mouse model of AD [92]. In this work, Ostapchenko et al. [93] showed that the knockout of TRPM2 reduces endoplasmic reticulum stress, age-dependent spatial memory deficit and microglia activation in APP/PS1 mice following Aβ treatment, although TRPM2^−/−^/APP/PS1 transgenic mice did not show any change in plaques formation. These results suggest that the deletion of TRPM2 channel is protective in AD, which may be achieved through the activation of microglia. Indeed, TRPM2 participates in the neuroinflammation induced by Aβ through microglia activation and generation of TNFα in a pathway, which involves ROS activation of poly (ADP-ribose) polymerase-1 (PARP-1) [94] (Figure 2).

Finally, TRPM2 has been proposed to contribute to cerebrovascular dysfunction in AD following the channel activation in vascular endothelial cells by Aβ [95]. These authors have demonstrated in different AD models that the cerebrovascular dysfunction requires oxidative stress-induced PARP-1 activity and even depends on TRPM2 activation in cerebral endothelial cells, which results in increased intracellular Ca^2+^. These findings highlight the possibility that endothelial TRPM2 channels may be a potential therapeutic target to counteract the cerebrovascular effects of Aβ.

TRPM2 has been also associated with bipolar disorder. Patients with Type I bipolar disorder show high basal Ca^2+^ levels and a susceptibility locus on chromosome 21q22.3., which is a region containing the TRPM2 gene [96,97]. Although TRPM2 variants with a single amino substitution (e.g., Asp543Glu) have been detected in patients with bipolar disorder, the role of these variants in the pathogenesis of the bipolar disorder remains unknown. Alterations in TRPM2 channel expression and function have also been demonstrated in other chronic diseases, such as Western Pacific amyotrophic lateral sclerosis (ALS) and PD. Intriguingly, Hermosura et al. have identified a mutation (P1018L) in ALS and PD patients, which is located in the pore loop of the TRPM2 channel and induces rapid channel inactivation [98].

### 4.2. Acute Neurodegenerative Diseases

Acute neuronal damage involves several processes, including excitotoxicity, inflammation, necrosis and apoptosis. In general, focal impairment of cerebral blood flow diminishes the delivery of substrates and impairs the maintenance of ionic gradients. As a consequence, dendritic and presynaptic voltage-dependent Ca^2+^ channels are activated and excitatory amino acids are released into the extracellular space. This starts a process called “excitotoxicity”, which represents the most important mechanism of cell death in stroke, CNS trauma and epilepsy [99]. Pharmacological blockade of glutamate receptors can prevent excitotoxicity and may have a neuroprotective effect, as demonstrated in animal models of stroke [100]. Although glutamate excitotoxicity is the main mechanism involved in neuronal death in CI, the significant contribution of Ca^2+^-permeable non-selective cation channels has also been demonstrated [20]. TRPM2 activation has been linked to cell death, suggesting that the channel can be considered as a key downstream player of several signaling pathways mediating cell death in response to CI and reperfusion injury [101]. Indeed, in a rat model of ischemia, the expression of TRPM2 mRNA increased from 1 to 4 weeks following stroke induction. The increased expression of TRPM2 was related to its transcriptional upregulation in glial cells in response to oxidative stress, which resulted in the promotion of cytokine release, exacerbation of inflammation and initiation of neuronal death [46]. Thus, following transient ischemia, the TRPM2 expressed in glia may be involved in the consequent injury [102].

**Figure 3:**
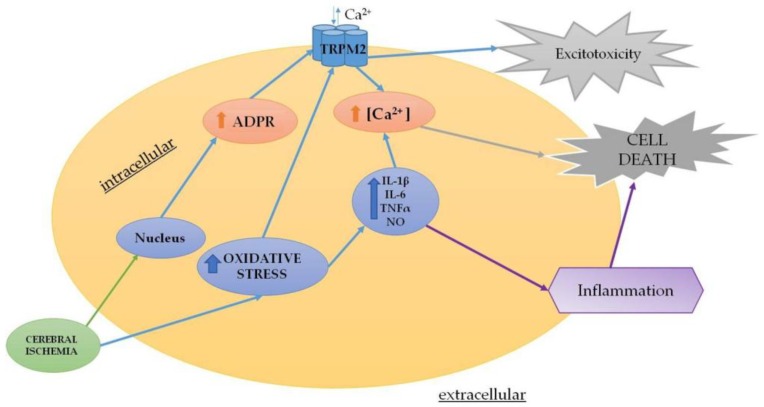
Summary of the main mechanisms modulated by TRPM2 in acute neurodegenerative diseases.

Similarly, several studies have highlighted the link between ischemic neuronal death and TRPM2 activation [103,104]. A key source of ROS in the brain is produced by microglia and astrocytes. In this scenario, ROS may either directly or indirectly induce neuronal cell death or may increase glial proliferation to protect neurons. A wide range of stimuli activates microglia and the effects of activating factors are partially mediated by the modulation of intracellular Ca^2+^ [102]. In summary, TRPM2 may be considered as the connection point between Ca^2+^- and ROS-dependent signaling pathways [105] (Figure 3). In this context, Jia et al. [106] showed both in vitro and in vivo that TRPM2 inhibition or knockdown is neuroprotective against CI. Indeed, the pharmacological inhibitors and RNA interference targeting TRPM2 reduced the infarct volume in vivo and decreased neuronal cell death in vitro. In 2006, Fonfria et al. demonstrated that the up-regulation of TRPM2 correlates with microglial activation in a rat model of stroke [102]. Authors reported that the up-regulation of TRPM2 is associated over time with microglial activation. They also demonstrated that TRPM2 plays a key role in the stress-induced activation of these cells in rat primary microglia.

Interestingly, Shimizu et al. demonstrated the important contribution of TRPM2 in CI, especially in males, suggesting a sex difference in the role of TRPM2 in ischemic cell death [107]. To date, the patients with stroke are extremely challenging to treat, which is possibly due to the lack of knowledge about the sex difference in this disease [108,109]. The most important and non-modifiable risk factors for stroke are age and gender and hence, Shimizu et al. focused on a membrane-permeable selective inhibitor of TRPM2 that can be used to understand its role in neuronal injury following focal CI in aged mice of both sexes [61]. Moreover, Jia et al. showed that the TRPM2 inhibitor clotrimazole reduces hippocampal CA1 neuronal injury in male mice and may be a potential neuroprotective agent against ischemic damage [106]. Thus, the gender specific effect of TRPM2 inhibition in focal CI is of particular interest, considering the different vulnerability of sexes to CI in the human population [110].

## 5. Future Perspectives

In conclusion, we have shown multiple aspects of TRPM2 in the human body, especially in the brain. The versatile and intriguing nature of TRPM2 have made it one of the most fascinating ion channels in our body. The role of TRPM2 in health and disease is becoming increasingly relevant. We have to emphasize that the future use of drugs that are able to block TRPM2 function has raised some concerns about the potential side effects. As we discussed in this review, the role of TRPM2 in a plethora of physiological processes, such as insulin release, temperature sensation-regulation and immune function, inevitably needs to be considered in any study or development of new therapeutic strategies. Hopefully, extensive research on pharmacological tools will aid in characterizing TRPM2 and will be essential to develop selective neuroprotective strategies in order to counteract neurodegeneration and to slow down the aging process and improve the quality of life in the elderly population.

## References

[1] Wu L.-J., Sweet T.-B., Clapham D.E. (2010). International Union of Basic and Clinical Pharmacology. LXXVI. Current Progress in the Mammalian TRP Ion Channel Family. Pharmacol. Rev..

[2] Gaudet R. (2009). Divide and conquer: High resolution structural information on TRP channel fragments. J. Gen. Physiol..

[3] Nilius B., Szallasi A. (2014). Transient Receptor Potential Channels as Drug Targets: From the Science of Basic Research to the Art of Medicine. Pharmacol. Rev..

[4] Gees M., Colsoul B., Nilius B. (2010). The role of transient receptor potential cation channels in Ca^2+^ signaling. Cold Spring Harb. Perspect. Biol..

[5] Nieto-Posadas A., Jara-Oseguera A., Rosenbaum T. (2011). TRP channel gating physiology. Curr. Top. Med. Chem..

[6] Jara-Oseguera A., Islas L.D. (2013). The role of allosteric coupling on thermal activation of thermo-TRP channels. Biophys. J..

[7] Ramsey I.S., Delling M., Clapham D.E. (2006). An introduction to TRP channels. Annu. Rev. Physiol..

[8] Clapham D.E. (1995). Calcium signaling. Cell.

[9] Clapham D.E., Montell C., Schultz G., Julius D. (2003). International Union of Pharmacology. XLIII. Compendium of voltage-gated ion channels: Transient receptor potential channels. Pharmacol. Rev..

[10] Clapham D.E. (2003). TRP channels as cellular sensors. Nature.

[11] Aarts M.M., Tymianski M. (2005). TRPMs and neuronal cell death. Pflugers Arch. Eur. J. Physiol..

[12] Arundine M., Tymianski M. (2003). Molecular mechanisms of calcium-dependent neurodegeneration in excitotoxicity. Cell Calcium.

[13] Choi D.W. (1988). Calcium-mediated neurotoxicity: Relationship to specific channel types and role in ischemic damage. Trends Neurosci..

[14] Vriens J., Watanabe H., Janssens A., Droogmans G., Voets T., Nilius B. (2004). Cell swelling, heat, and chemical agonists use distinct pathways for the activation of the cation channel TRPV4. Proc. Natl. Acad. Sci. USA.

[15] Sawamura S., Shirakawa H., Nakagawa T., Mori Y., Kaneko S. (2017). TRP Channels in the Brain: What Are They There For?. Neurobiology of TRP Channels.

[16] Grigoryan G., Keating A.E. (2008). Structural specificity in coiled-coil interactions. Curr. Opin. Struct. Biol..

[17] Fonfria E., Murdock P.R., Cusdin F.S., Benham C.D., Kelsell R.E., McNulty S. (2006). Tissue distribution profiles of the human TRPM cation channel family. J. Recept. Signal Transduct. Res..

[18] Nagamine K., Kudoh J., Minoshima S., Kawasaki K., Asakawa S., Ito F., Shimizu N. (1998). Molecular cloning of a novel putative Ca^2+^ channel protein (TRPC7) highly expressed in brain. Genomics.

[19] Xia R., Mei Z.-Z., Mao H.-J., Yang W., Dong L., Bradley H., Beech D.J., Jiang L.-H. (2008). Identification of pore residues engaged in determining divalent cationic permeation in transient receptor potential melastatin subtype channel 2. J. Biol. Chem..

[20] Belrose J.C., Jackson M.F. (2018). TRPM2: A candidate therapeutic target for treating neurological diseases. Acta Pharmacol. Sin..

[21] Yue Z., Xie J., Yu A.S., Stock J., Du J., Yue L. (2015). Role of TRP channels in the cardiovascular system. Am. J. Physiol. Circ. Physiol..

[22] Kheradpezhouh E., Ma L., Morphett A., Barritt G.J., Rychkov G.Y. (2014). TRPM2 channels mediate acetaminophen-induced liver damage. Proc. Natl. Acad. Sci. USA.

[23] Zhang Z., Tóth B., Szollosi A., Chen J., Csanády L. (2018). Structure of a TRPM2 channel in complex with Ca^2+^ explains unique gating regulation. Elife.

[24] Turlova E., Feng Z., Sun H. (2018). The role of TRPM2 channels in neurons, glial cells and the blood-brain barrier in cerebral ischemia and hypoxia. Acta Pharmacol. Sin..

[25] Wehage E., Eisfeld J., Heiner I., Jüngling E., Zitt C., Lückhoff A. (2002). Activation of the cation channel long transient receptor potential channel 2 (LTRPC2) by hydrogen peroxide. A splice variant reveals a mode of activation independent of ADP-ribose. J. Biol. Chem..

[26] McHugh D., Flemming R., Xu S.-Z., Perraud A.-L., Beech D.J. (2003). Critical intracellular Ca^2+^ dependence of transient receptor potential melastatin 2 (TRPM2) cation channel activation. J. Biol. Chem..

[27] Perraud A.L., Fleig A., Dunn C.A., Bagley L.A., Launay P., Schmitz C., Stokes A.J., Zhu Q., Bessman M.J., Penner R. (2001). ADP-ribose gating of the calcium-permeable LTRPC2 channel revealed by Nudix motif homology. Nature.

[28] Fliegert R., Bauche A., Wolf Pérez A.-M., Watt J.M., Rozewitz M.D., Winzer R., Janus M., Gu F., Rosche A., Harneit A. (2017). 2′-Deoxyadenosine 5′-diphosphoribose is an endogenous TRPM2 superagonist. Nat. Chem. Biol..

[29] Harteneck C. (2005). Function and pharmacology of TRPM cation channels. Naunyn Schmiedebergs Arch. Pharmacol..

[30] Fonfria E., Marshall I.C.B., Benham C.D., Boyfield I., Brown J.D., Hill K., Hughes J.P., Skaper S.D., McNulty S. (2004). TRPM2 channel opening in response to oxidative stress is dependent on activation of poly(ADP-ribose) polymerase. Br. J. Pharmacol..

[31] Olah M.E., Jackson M.F., Li H., Perez Y., Sun H.-S., Kiyonaka S., Mori Y., Tymianski M., MacDonald J.F. (2009). Ca^2+^-dependent induction of TRPM2 currents in hippocampal neurons. J. Physiol..

[32] Xie Y.-F., Belrose J.C., Lei G., Tymianski M., Mori Y., MacDonald J.F., Jackson M.F. (2011). Dependence of NMDA/GSK-3β Mediated Metaplasticity on TRPM2 Channels at Hippocampal CA3-CA1 Synapses. Mol. Brain.

[33] Jang Y., Lee M.H., Lee J., Jung J., Lee S.H., Yang D.-J., Kim B.W., Son H., Lee B., Chang S. (2014). TRPM2 mediates the lysophosphatidic acid-induced neurite retraction in the developing brain. Pflügers Arch. Eur. J. Physiol..

[34] Yamamoto S., Shimizu S., Kiyonaka S., Takahashi N., Wajima T., Hara Y., Negoro T., Hiroi T., Kiuchi Y., Okada T. (2008). TRPM2-mediated Ca^2+^ influx induces chemokine production in monocytes that aggravates inflammatory neutrophil infiltration. Nat. Med..

[35] Haraguchi K., Kawamoto A., Isami K., Maeda S., Kusano A., Asakura K., Shirakawa H., Mori Y., Nakagawa T., Kaneko S. (2012). TRPM2 contributes to inflammatory and neuropathic pain through the aggravation of pronociceptive inflammatory responses in mice. J. Neurosci..

[36] Kraft R., Grimm C., Grosse K., Hoffmann A., Sauerbruch S., Kettenmann H., Schultz G., Harteneck C. (2004). Hydrogen peroxide and ADP-ribose induce TRPM2-mediated calcium influx and cation currents in microglia. Am. J. Physiol. Cell Physiol..

[37] Miyake T., Shirakawa H., Kusano A., Sakimoto S., Konno M., Nakagawa T., Mori Y., Kaneko S. (2014). TRPM2 contributes to LPS/IFNγ-induced production of nitric oxide via the p38/JNK pathway in microglia. Biochem. Biophys. Res. Commun..

[38] Hiroi T., Wajima T., Negoro T., Ishii M., Nakano Y., Kiuchi Y., Mori Y., Shimizu S. (2013). Neutrophil TRPM2 channels are implicated in the exacerbation of myocardial ischaemia/reperfusion injury. Cardiovasc. Res..

[39] Tripathi J.K., Sharma A., Sukumaran P., Sun Y., Mishra B.B., Singh B.B., Sharma J. (2018). Oxidant sensor cation channel TRPM2 regulates neutrophil extracellular trap formation and protects against pneumoseptic bacterial infection. FASEB J..

[40] Zou J., Ainscough J.F., Yang W., Sedo A., Yu S.-P., Mei Z.-Z., Sivaprasadarao A., Beech D.J., Jiang L.-H. (2013). A differential role of macrophage TRPM2 channels in Ca^2+^ signaling and cell death in early responses to H_2_O_2_. Am. J. Physiol. Cell Physiol..

[41] Di A., Kiya T., Gong H., Gao X., Malik A.B. (2017). Role of the phagosomal redox-sensitive TRP channel TRPM2 in regulating bactericidal activity of macrophages. J. Cell Sci..

[42] Roedding A.S., Gao A.F., Au-Yeung W., Scarcelli T., Li P.P., Warsh J.J. (2012). Effect of oxidative stress on TRPM2 and TRPC3 channels in B lymphoblast cells in bipolar disorder. Bipolar Disord..

[43] Syed Mortadza S.A., Wang L., Li D., Jiang L.-H. (2015). TRPM2 Channel-Mediated ROS-Sensitive Ca^2+^ Signaling Mechanisms in Immune Cells. Front. Immunol..

[44] Wehrhahn J., Kraft R., Harteneck C., Hauschildt S. (2010). Transient receptor potential melastatin 2 is required for lipopolysaccharide-induced cytokine production in human monocytes. J. Immunol..

[45] Sumoza-Toledo A., Lange I., Cortado H., Bhagat H., Mori Y., Fleig A., Penner R., Partida-Sánchez S. (2011). Dendritic cell maturation and chemotaxis is regulated by TRPM2-mediated lysosomal Ca^2+^ release. FASEB J..

[46] Faouzi M., Penner R. (2014). Mammalian Transient Receptor Potential (TRP) Cation Channels. Handbook of Experimental Pharmacology.

[47] Knowles H., Heizer J.W., Li Y., Chapman K., Ogden C.A., Andreasen K., Shapland E., Kucera G., Mogan J., Humann J. (2011). Transient Receptor Potential Melastatin 2 (TRPM2) ion channel is required for innate immunity against Listeria monocytogenes. Proc. Natl. Acad. Sci. USA.

[48] Di A., Gao X.-P., Qian F., Kawamura T., Han J., Hecquet C., Ye R.D., Vogel S.M., Malik A.B. (2011). The redox-sensitive cation channel TRPM2 modulates phagocyte ROS production and inflammation. Nat. Immunol..

[49] Kashio M., Sokabe T., Shintaku K., Uematsu T., Fukuta N., Kobayashi N., Mori Y., Tominaga M. (2012). Redox signal-mediated sensitization of transient receptor potential melastatin 2 (TRPM2) to temperature affects macrophage functions. Proc. Natl. Acad. Sci. USA.

[50] Togashi K., Hara Y., Tominaga T., Higashi T., Konishi Y., Mori Y., Tominaga M. (2006). TRPM2 activation by cyclic ADP-ribose at body temperature is involved in insulin secretion. EMBO J..

[51] Uchida K., Dezaki K., Damdindorj B., Inada H., Shiuchi T., Mori Y., Yada T., Minokoshi Y., Tominaga M. (2011). Lack of TRPM2 impaired insulin secretion and glucose metabolisms in mice. Diabetes.

[52] Pang B., Kim S., Li D., Ma Z., Sun B., Zhang X., Wu Z., Chen L. (2017). Glucagon-like peptide-1 potentiates glucose-stimulated insulin secretion via the transient receptor potential melastatin 2 channel. Exp. Ther. Med..

[53] Tan C.-H., McNaughton P.A. (2016). The TRPM2 ion channel is required for sensitivity to warmth. Nature.

[54] Tan C.-H., McNaughton P.A. (2018). TRPM2 and warmth sensation. Pflügers Arch. Eur. J. Physiol..

[55] Song K., Wang H., Kamm G.B., Pohle J., de Castro Reis F., Heppenstall P., Wende H., Siemens J. (2016). The TRPM2 channel is a hypothalamic heat sensor that limits fever and can drive hypothermia. Science.

[56] Kashio M., Tominaga M. (2017). The TRPM2 channel: A thermo-sensitive metabolic sensor. Channels.

[57] Sun L., Yau H.-Y., Wong W.-Y., Li R.A., Huang Y., Yao X. (2012). Role of TRPM2 in H(2)O(2)-induced cell apoptosis in endothelial cells. PLoS ONE.

[58] Massullo P., Sumoza-Toledo A., Bhagat H., Partida-Sánchez S. (2006). TRPM channels, calcium and redox sensors during innate immune responses. Semin. Cell Dev. Biol..

[59] Barbosa M., Valentão P., Andrade P.B. (2014). Bioactive compounds from macroalgae in the new millennium: Implications for neurodegenerative diseases. Mar. Drugs.

[60] Thibault O., Gant J.C., Landfield P.W. (2007). Expansion of the calcium hypothesis of brain aging and Alzheimer’s disease: Minding the store. Aging Cell.

[61] Shimizu T., Dietz R.M., Cruz-Torres I., Strnad F., Garske A.K., Moreno M., Venna V.R., Quillinan N., Herson P.S. (2016). Extended therapeutic window of a novel peptide inhibitor of TRPM2 channels following focal cerebral ischemia. Exp. Neurol..

[62] Takahashi N., Kozai D., Kobayashi R., Ebert M., Mori Y. (2011). Roles of TRPM2 in oxidative stress. Cell Calcium.

[63] Langley B., Ratan R.R. (2004). Oxidative stress-induced death in the nervous system: Cell cycle dependent or independent?. J. Neurosci. Res..

[64] Chandra J., Samali A., Orrenius S. (2000). Triggering and modulation of apoptosis by oxidative stress. Free Radic. Biol. Med..

[65] Chung W.-S., Welsh C.A., Barres B.A., Stevens B. (2015). Do glia drive synaptic and cognitive impairment in disease?. Nat. Neurosci..

[66] Urrutia P.J., Hirsch E.C., González-Billault C., Núñez M.T. (2017). Hepcidin attenuates amyloid beta-induced inflammatory and pro-oxidant responses in astrocytes and microglia. J. Neurochem..

[67] Daulatzai M.A. (2016). Fundamental role of pan-inflammation and oxidative-nitrosative pathways in neuropathogenesis of Alzheimer’s disease in focal cerebral ischemic rats. Am. J. Neurodegener. Dis..

[68] Mattson M.P. (2007). Calcium and neurodegeneration. Aging Cell.

[69] Nikoletopoulou V., Tavernarakis N. (2012). Calcium homeostasis in aging neurons. Front. Genet..

[70] Kregel K.C., Zhang H.J. (2007). An integrated view of oxidative stress in aging: Basic mechanisms, functional effects, and pathological considerations. Am. J. Physiol. Regul. Integr. Comp. Physiol..

[71] Parihar M.S., Kunz E.A., Brewer G.J. (2008). Age-related decreases in NAD(P)H and glutathione cause redox declines before ATP loss during glutamate treatment of hippocampal neurons. J. Neurosci. Res..

[72] Cooper A.J., Kristal B.S. (1997). Multiple roles of glutathione in the central nervous system. Biol. Chem..

[73] Belrose J.C., Xie Y.-F., Gierszewski L.J., MacDonald J.F., Jackson M.F. (2012). Loss of glutathione homeostasis associated with neuronal senescence facilitates TRPM2 channel activation in cultured hippocampal pyramidal neurons. Mol. Brain.

[74] Robillard J.M., Gordon G.R., Choi H.B., Christie B.R., MacVicar B.A. (2011). Glutathione restores the mechanism of synaptic plasticity in aged mice to that of the adult. PLoS ONE.

[75] Aoyama K., Suh S.W., Hamby A.M., Liu J., Chan W.Y., Chen Y., Swanson R.A. (2006). Neuronal glutathione deficiency and age-dependent neurodegeneration in the EAAC1 deficient mouse. Nat. Neurosci..

[76] Norris C.M., Korol D.L., Foster T.C. (1996). Increased susceptibility to induction of long-term depression and long-term potentiation reversal during aging. J. Neurosci..

[77] Xie Y.F., MacDonald J.F., Jackson M.F. (2010). TRPM2, calcium and neurodegenerative diseases. Int. J. Physiol. Pathophysiol. Pharmacol..

[78] Butterfield D., Drake J., Pocernich C., Castegna A. (2001). Evidence of oxidative damage in Alzheimer’s disease brain: Central role for amyloid β-peptide. Trends Mol. Med..

[79] Balaban H., Nazıroğlu M., Demirci K., Övey İ.S. (2017). The Protective Role of Selenium on Scopolamine-Induced Memory Impairment, Oxidative Stress, and Apoptosis in Aged Rats: The Involvement of TRPM2 and TRPV1 Channels. Mol. Neurobiol..

[80] Frozza R.L., Bernardi A., Hoppe J.B., Meneghetti A.B., Matté A., Battastini A.M.O., Pohlmann A.R., Guterres S.S., Salbego C. (2013). Neuroprotective effects of resveratrol against Aβ administration in rats are improved by lipid-core nanocapsules. Mol. Neurobiol..

[81] Morroni F., Sita G., Tarozzi A., Rimondini R., Hrelia P. (2016). Early effects of Aβ1-42 oligomers injection in mice: Involvement of PI3K/Akt/GSK3 and MAPK/ERK1/2 pathways. Behav. Brain Res..

[82] Jo S., Yarishkin O., Hwang Y.J., Chun Y.E., Park M., Woo D.H., Bae J.Y., Kim T., Lee J., Chun H. (2014). GABA from reactive astrocytes impairs memory in mouse models of Alzheimer’s disease. Nat. Med..

[83] Pascale A., Etcheberrigaray R. (1999). Calcium alterations in Alzheimer’s disease: Pathophysiology, models and therapeutic opportunities. Pharmacol. Res..

[84] Kurz A.F. (2005). Uncommon neurodegenerative causes of dementia. Int. Psychogeriatr..

[85] Nishimura I., Takazaki R., Kuwako K.I., Enokido Y., Yoshikawa K. (2003). Upregulation and antiapoptotic role of endogenous Alzheimer amyloid precursor protein in dorsal root ganglion neurons. Exp. Cell Res..

[86] Nazıroğlu M. (2011). TRPM2 cation channels, oxidative stress and neurological diseases: Where are we now?. Neurochem. Res..

[87] Saharan S., Mandal P.K. (2014). The emerging role of glutathione in Alzheimer’s disease. J. Alzheimers Dis..

[88] Lesuisse C., Martin L.J. (2002). Long-term culture of mouse cortical neurons as a model for neuronal development, aging, and death. J. Neurobiol..

[89] Övey İ.S., Naziroğlu M. (2015). Homocysteine and cytosolic GSH depletion induce apoptosis and oxidative toxicity through cytosolic calcium overload in the hippocampus of aged mice: Involvement of TRPM2 and TRPV1 channels. Neuroscience.

[90] Wang J., Jackson M.F., Xie Y.F. (2016). Glia and TRPM2 Channels in Plasticity of Central Nervous System and Alzheimer’s Diseases. Neural Plast..

[91] Fonfria E., Marshall I.C.B., Boyfield I., Skaper S.D., Hughes J.P., Owen D.E., Zhang W., Miller B.A., Benham C.D., McNulty S. (2005). Amyloid beta-peptide(1-42) and hydrogen peroxide-induced toxicity are mediated by TRPM2 in rat primary striatal cultures. J. Neurochem..

[92] Yankner B.A., Lu T., Loerch P. (2008). The aging brain. Annu. Rev. Pathol..

[93] Ostapchenko V.G., Chen M., Guzman M.S., Xie Y.-F., Lavine N., Fan J., Beraldo F.H., Martyn A.C., Belrose J.C., Mori Y. (2015). The Transient Receptor Potential Melastatin 2 (TRPM2) Channel Contributes to -Amyloid Oligomer-Related Neurotoxicity and Memory Impairment. J. Neurosci..

[94] Alawieyah Syed Mortadza S., Sim J.A., Neubrand V.E., Jiang L.-H. (2018). A critical role of TRPM2 channel in Aβ42-induced microglial activation and generation of tumor necrosis factor-α. Glia.

[95] Park L., Wang G., Moore J., Girouard H., Zhou P., Anrather J., Iadecola C. (2014). The key role of transient receptor potential melastatin-2 channels in amyloid-β-induced neurovascular dysfunction. Nat. Commun..

[96] Xu C., Macciardi F., Li P.P., Yoon I.-S., Cooke R.G., Hughes B., Parikh S.V., McIntyre R.S., Kennedy J.L., Warsh J.J. (2006). Association of the putative susceptibility gene, transient receptor potential protein melastatin type 2, with bipolar disorder. Am. J. Med. Genet. B Neuropsychiatr. Genet..

[97] McQuillin A., Bass N.J., Kalsi G., Lawrence J., Puri V., Choudhury K., Detera-Wadleigh S.D., Curtis D., Gurling H.M.D. (2006). Fine mapping of a susceptibility locus for bipolar and genetically related unipolar affective disorders, to a region containing the C21ORF29 and TRPM2 genes on chromosome 21q22.3. Mol. Psychiatry.

[98] Hermosura M.C., Cui A.M., Go R.C.V., Davenport B., Shetler C.M., Heizer J.W., Schmitz C., Mocz G., Garruto R.M., Perraud A.-L. (2008). Altered functional properties of a TRPM2 variant in Guamanian ALS and PD. Proc. Natl. Acad. Sci. USA.

[99] Olney J.W. (1969). Brain lesions, obesity, and other disturbances in mice treated with monosodium glutamate. Science.

[100] Dugan L.L., Choi D.W. (1994). Excitotoxicity, free radicals, and cell membrane changes. Ann. Neurol..

[101] MacDonald J.F., Xiong Z.-G., Jackson M.F. (2006). Paradox of Ca^2+^ signaling, cell death and stroke. Trends Neurosci..

[102] Fonfria E., Mattei C., Hill K., Brown J.T., Randall A., Benham C.D., Skaper S.D., Campbell C.A., Crook B., Murdock P.R. (2006). TRPM2 is elevated in the tMCAO stroke model, transcriptionally regulated, and functionally expressed in C13 microglia. J. Recept. Signal Transduct..

[103] Huang S., Turlova E., Li F., Bao M., Szeto V., Wong R., Abussaud A., Wang H., Zhu S., Gao X. (2017). Transient receptor potential melastatin 2 channels (TRPM2) mediate neonatal hypoxic-ischemic brain injury in mice. Exp. Neurol..

[104] Yamamoto S., Shimizu S. (2016). Targeting TRPM2 in ROS-Coupled Diseases. Pharmaceuticals.

[105] Hara Y., Wakamori M., Ishii M., Maeno E., Nishida M., Yoshida T., Yamada H., Shimizu S., Mori E., Kudoh J. (2002). LTRPC2 Ca^2+^-permeable channel activated by changes in redox status confers susceptibility to cell death. Mol. Cell.

[106] Jia J., Verma S., Nakayama S., Quillinan N., Grafe M.R., Hurn P.D., Herson P.S. (2011). Sex differences in neuroprotection provided by inhibition of TRPM2 channels following experimental stroke. J. Cereb. Blood Flow Metab..

[107] Shimizu K., Quillinan N., Orfila J.E., Herson P.S. (2016). Sirtuin-2 mediates male specific neuronal injury following experimental cardiac arrest through activation of TRPM2 ion channels. Exp. Neurol..

[108] Romero J.R., Morris J., Pikula A. (2008). Stroke prevention: Modifying risk factors. Ther. Adv. Cardiovasc. Dis..

[109] Herson P.S., Traystman R.J. (2014). Animal models of stroke: Translational potential at present and in 2050. Future Neurol..

[110] Wigginton J.G., Pepe P.E., Bedolla J.P., DeTamble L.A., Atkins J.M. (2002). Sex-related differences in the presentation and outcome of out-of-hospital cardiopulmonary arrest: A multiyear, prospective, population-based study. Crit. Care Med..

